# Increased Production of Abdominal Donor Site Fluid Following Microsurgical Breast Reconstruction With Superficial Inferior Epigastric Artery Versus Deep Inferior Epigastric Artery Perforator Flaps

**DOI:** 10.7759/cureus.38942

**Published:** 2023-05-12

**Authors:** Calum H Carslaw, Havish Samudrala, James Kerrison, Jack E Brooker, Nicholas G Rabey, Charles M Malata

**Affiliations:** 1 School of Clinical Medicine, University of Cambridge, Cambridge, GBR; 2 Department of Intensive Care, Royal Papworth Hospital NHS Foundation Trust, Cambridge, GBR; 3 Department of Plastic and Reconstructive Surgery, Cambridge University Hospitals NHS Foundation Trust, Addenbrooke's Hospital, Cambridge, GBR; 4 School of Medicine, Anglia Ruskin University, Cambridge, GBR; 5 Department of Plastic and Reconstructive Surgery, and Cambridge Breast Unit, Cambridge University Hospitals NHS Foundation Trust, Addenbrooke's Hospital, Cambridge, GBR

**Keywords:** donor site morbidity, microvascular surgery, seroma, diep flaps, siea flaps, breast reconstruction

## Abstract

Introduction and aims

Donor site seroma following abdominal flap harvest for breast reconstruction is common in both deep inferior epigastric artery perforator (DIEP) and superficial inferior epigastric artery (SIEA) flaps. We tested the hypothesis that there is increased donor site fluid following SIEA dissection compared to DIEP.

Materials and methods

Of60 SIEA breast reconstructions performed by one surgeon in 50 patients (2004-2019), complete data were available for 31 patients. Eighteen unilateral SIEAs were matched with 18 unilateral DIEPs. Thirteen bilateral flap harvests involving an SIEA were matched with 13 bilateral DIEP controls. Their cumulative abdominal drain outputs, times to drain removal, hospital stay, and number and volume of seroma aspirations were compared.

Results

Patients who underwent an SIEA flap harvest had significantly increased drain output compared to only a DIEP flap harvest (SIEA=1,078 mL, DIEP=500 mL, p<0.001), which remained significant after controlling for confounding variables (p=0.002). There was increased time until drain removal (SIEA=11 days, DIEP=6 days, p=0.010), and patients who underwent an SIEA harvest were 14 times more likely to be discharged with a drain in situ (odds ratio (OR)=14.6, 95% confidence interval (CI)=2.8203-75.9565, p=0.0014). There was no significant difference in the number or volume of outpatient aspirations, length of hospital admission, or total seroma volume.

Conclusion

This study demonstrated that SIEA harvest is a significant predictor of increased abdominal drain output postoperatively. This accounted for longer periods before drain removal and more patients discharged with an abdominal drain in situ and should be an important consideration for reconstructive surgeons. There was no demonstrable difference in the number or volume of seroma aspirations after drain removal for either group.

## Introduction

Breast reconstruction following mastectomy forms an important part of holistic care following breast cancer diagnosis and treatment. Reconstruction using autologous flaps most frequently utilizes abdominal tissue, and the two main types of total muscle-sparing flaps used involve the deep inferior epigastric artery perforator (DIEP) and superficial inferior epigastric artery (SIEA). DIEP flap harvest entails splitting the rectus sheath and muscle to locate and dissect the perforating artery and veins back to the flap pedicle, whereas the SIEA flap involves harvesting the superficial vascular pedicle of the lower abdominal pannus without breaching the sheath or muscle. The DIEP flap is more commonly carried out because of the greater likelihood of finding adequately sized vessels for anastomosis [1,2]. Consequently, there is a reduced need for anastomosis revision required with DIEP flaps and possibly a lower rate of hematoma formation [3]. As the SIEA flap involves no dissection of the anterior abdominal muscular wall or fascia, there is reduced donor site morbidity [4-7]. The SIEA flap vasculature is, however, less predictable and therefore not as commonly used. However, it is the abdominal flap of choice if its vessels, especially the artery, are found to be adequate during flap harvest [7-12]. Typically, the superficial inferior epigastric artery is only dissected after confirming the artery is of sufficient size and pulsatile when dissecting the DIEP pedicle. A large multicenter study showed that SIEA flaps were associated with higher BREAST-Q abdominal physical well-being scores compared with DIEP flaps at one year [6].

Donor site seroma is a commonly reported complication of DIEP and SIEA flaps [1,13-16]. The rate of seroma formation has been reported to be higher in SIEA flaps, and this is thought to be due to damage to the superficial abdominal wall lymphatic vessels upon harvesting the superficial inferior epigastric artery as they drain to the groin lymph nodes [13,17,18]. Allen and Heitland (2002) [1] noted a greater seroma rate in SIEA flaps, but this was not quantified. Moradi et al. (2011) [18] studied a small number of patients over a 10-month period and found that the seven SIEA flaps on average produced 2248 mL total abdominal fluid compared to 531 mL in the 28 comparator DIEP flaps. Additionally, a recent study by Erdmann-Sager et al. [6] in a review of 11 surgical centers showed a donor site seroma rate of 30% (19/62) in SIEA flap harvest compared with 7% (25/355) for DIEP flap harvest. All published reports to date, including the multicenter study, involve a small number of SIEA flaps or have assessed donor site fluid formation grossly (by a single seroma rate).

It was therefore the objective of this investigation to evaluate a single surgeon SIEA flap series over 15 years comparing various aspects of donor site seroma formation in SIEA and DIEP abdominal flaps used for breast reconstruction. We tested the hypothesis that in general more abdominal donor site fluid is produced following SIEA flap harvest than DIEP flap dissection.

This work was previously presented at the 19th Annual Academic Surgical Congress in Houston, Texas, on February 9, 2023.

## Materials and methods

Study population and definitions

**Figure 1:**
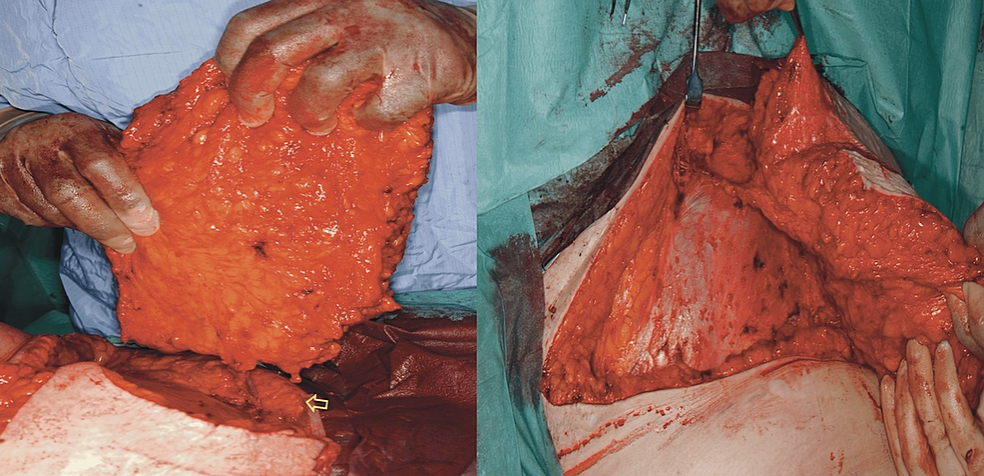
Intraoperative photograph of an SIEA flap This SIEA flap is held up to show the short vascular pedicle at the inferior end of the skin-fat flap prior to its division. The photograph on the right shows that there is no violation of the rectus sheath (shiny smooth appearance) let alone the muscle. The dissection of the SIEA pedicle extends deep into the groin and again emphasizes the short pedicle. SIEA: superficial inferior epigastric artery

**Figure 2:**
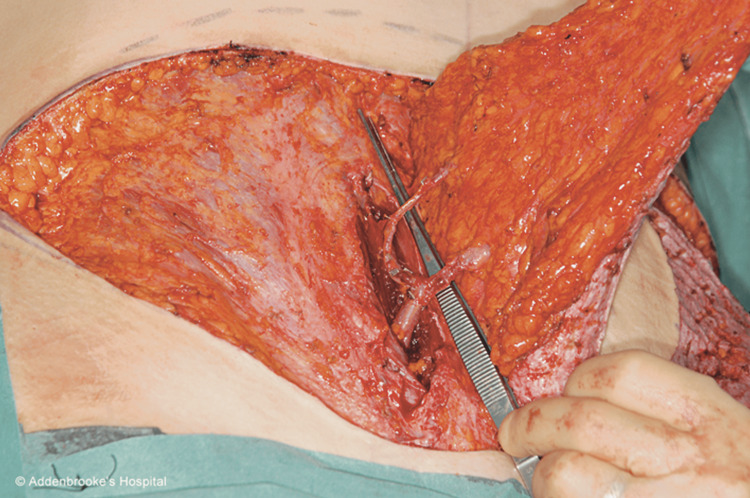
Intraoperative photograph of a right DIEP flap showing the main anatomical aspects of DIEP flap harvest There is a split in both the rectus sheath and muscle to allow dissection of the two medial row perforators and the main (flap) vascular pedicle from which they arise (caudally). There is no muscle sacrifice and often no need to expose the lateral border of the rectus muscle to access the vascular pedicle. This step is better at avoiding damage to the motor nerves. DIEP: deep inferior epigastric artery perforator

Patients who were operated on by the senior author (CMM) between 2004 and 2019 for breast reconstruction with free flaps were identified from the departmental free flap register and the surgeon’s logbook and reviewed for inclusion. Patients who received free transverse rectus abdominis myocutaneous (TRAM) or other free flaps at the same operation were excluded as were patients with incomplete drain output data. The decision to undertake an SIEA flap over a DIEP flap was often made intraoperatively rather than preoperatively based on the size and flow of the artery when visualized during DIEP dissection. Therefore, those patients who underwent DIEP flaps did not undergo dissection of the superficial inferior epigastric artery or vascular pedicle. The superficial inferior epigastric vein, which runs a different course to the artery and lymphatic vessels, was usually preserved to some length (Figure 1 and Figure 2).

Selection of controls/comparator group

**Figure 3:**
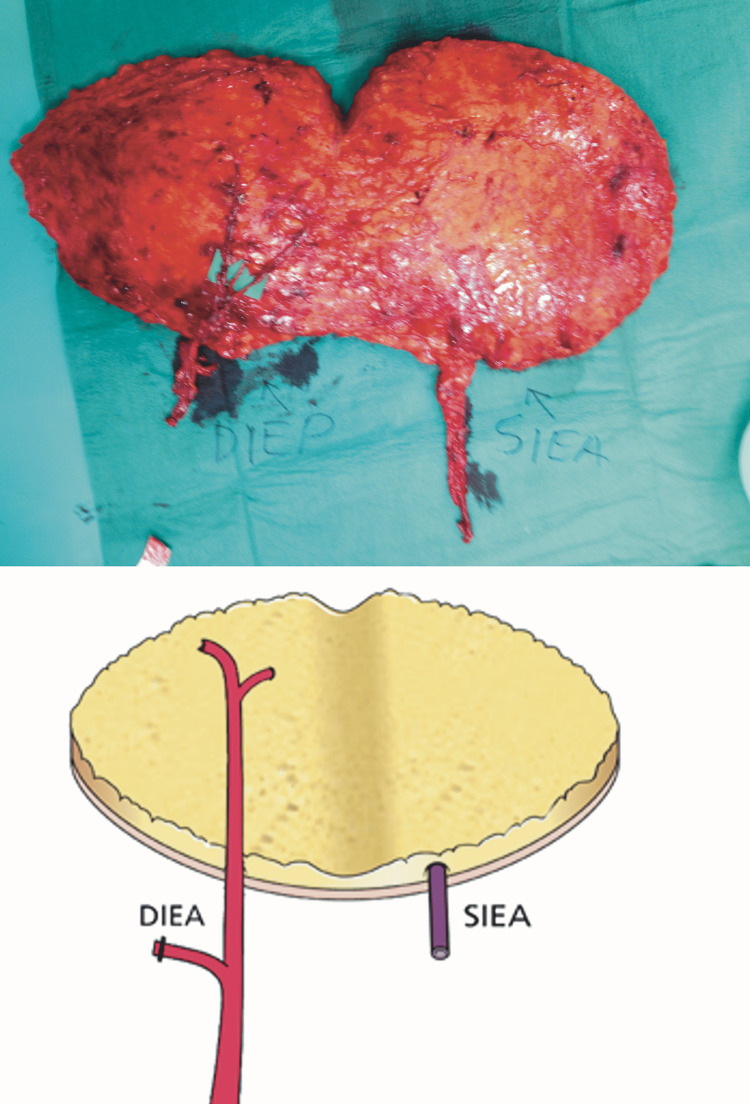
Intraoperative image of a DIEP-SIEA flap combination On the right is an SIEA flap with its short vascular pedicle, emphasizing the “tadpole” configuration. The left shows the DIEP vascular pedicle and three central row perforators. Its pedicle is longer and far more flexible and has the so-called “mushroom” configuration. DIEP: deep inferior epigastric artery perforator, SIEA: superficial inferior epigastric artery

Unilateral SIEA flaps (Figure 1) were paired with the nearest chronologically performed unilateral DIEP flap (Figure 2) to account for any effect of the surgical learning curve over the period. Bilateral SIEA-DIEP (Figure 3) and bilateral SIEA flaps were paired with the nearest chronologically performed bilateral DIEP-DIEP flap to account for the additional volume of seroma from the dissection of two vascular pedicles compared to one.

Data collection

Data were collected from both the paper records (first 10 years) and EPIC electronic medical records (from 2014). Variables collected were whether the flap involved SIEA or DIEP harvest, whether one or both sides of the abdomen were dissected, date of operation, patient age, total flap weight, smoking history, body mass index (BMI), length of hospital stay, total volume of abdominal drain output while in the hospital, drain duration, number of outpatient seroma aspirations, and volume of each seroma aspirated.

For categorization as SIEA or DIEP, the dissection of vessels alone was considered, independent of whether the vessels were subsequently used for anastomosis. The total flap weight was the total weight of flap tissue removed from the abdomen before flap trimming and shaping. Drain duration was defined as the number of days between the operation and removal of the last remaining abdominal drain. Inpatient drainage was collected from daily drain output charts within inpatient notes. For patients discharged with drains in situ, daily outputs were taken from patient-recorded values that were reported to the Plastic Surgery Dressings Unit. The total volume of abdominal fluid output was the sum of inpatient drainage amounts and seroma aspirations.

Ethics

The study was registered with the hospital’s audit and quality improvement department and adhered to the Strengthening the Reporting of Observational Studies in Epidemiology (STROBE) guidelines.

Statistical analysis

Statistical tests were carried out on Statistical Package for the Social Sciences (SPSS) version 28.0.1.1 (IBM SPSS Statistics, Armonk, NY, USA). Baseline characteristics were compared with a two-tailed t-test, and dependent variables were compared using the independent samples median test, and univariate regression analysis included independent variables of age, BMI, flap weight, and type of flap.

## Results

**Figure 4:**
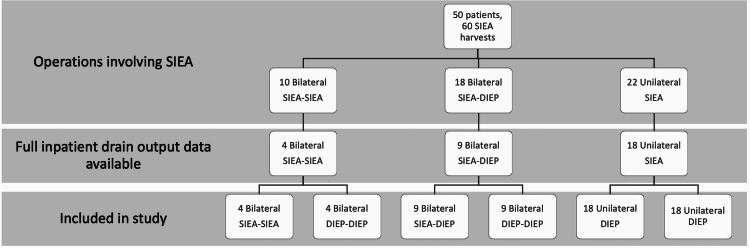
Flowchart showing total number of SIEA patients operated on by the senior author, and patients included in the study DIEP: deep inferior epigastric artery perforator, SIEA: superficial inferior epigastric artery

Fifty patients underwent operations involving SIEA vascular pedicle dissections during the study period comprising a total of 60 successful SIEA flaps. Of these, 31 operations involving SIEA had full drain output data and were compared to an appropriately matched DIEP control (Figure 4). Eighteen patients who underwent unilateral SIEA were matched with 18 patients who underwent unilateral DIEP harvest, while 13 patients who underwent bilateral SIEA harvests (nine SIEA-DIEP harvests and three SIEA-SIEA harvests) were matched with 13 bilateral DIEP controls.

Baseline characteristics for these patients, including median and range, are shown in Table 1. There was a statistically significant difference in the median flap weights (SIEA=1,020 g, DIEP=844.5 g, p=0.038) and BMI (SIEA=30 kg/m^2^, DIEP=27.1 kg/m^2^, p=0.001) between groups. The number of patients reporting each variable is shown in Table 2.

**Table 1:** Baseline characteristics (median (range)) of patients in the study, compared using a two-tailed t-test DIEP: deep inferior epigastric artery perforator, SIEA: superficial inferior epigastric artery, BMI: body mass index

	SIEA (n=31)	DIEP (n=31)	P-value
Age at operation (years)	51 (31-69)	52 (32-74)	0.979
BMI (kg/m^2^)	30.0 (23.8-38.7)	27.1 (19.4-33.8)	0.001*
Total flap weight (g)	1020 (593-2888)	844.5 (352-1969)	0.038*
Smoking history	0 smokers, 7 ex-smokers, 20 non-smokers, 4 not reported	2 smokers, 4 ex-smokers, 20 non-smokers, 5 not reported	

**Table 2:** Number of patients with each variable reported DIEP: deep inferior epigastric artery perforator, SIEA: superficial inferior epigastric artery, BMI: body mass index

	Unilateral SIEA	Unilateral DIEP	Bilateral DIEP-DIEP	Bilateral SIEA-DIEP	Bilateral SIEA-SIEA
Inpatient drain output data	18	18	13	9	4
Outpatient drain output data (if applicable)	18	18	13	9	4
Outpatient seroma aspiration	16	18	13	9	4
Day of drain removal	13	12	12	9	3
Length of hospital stay	18	18	13	9	4
Total flap weight	14	15	13	9	4
ΒΜΙ	15	18	11	9	4
Age at operation	18	18	13	9	4
Smoking status	14	13	13	9	4

**Figure 5:**
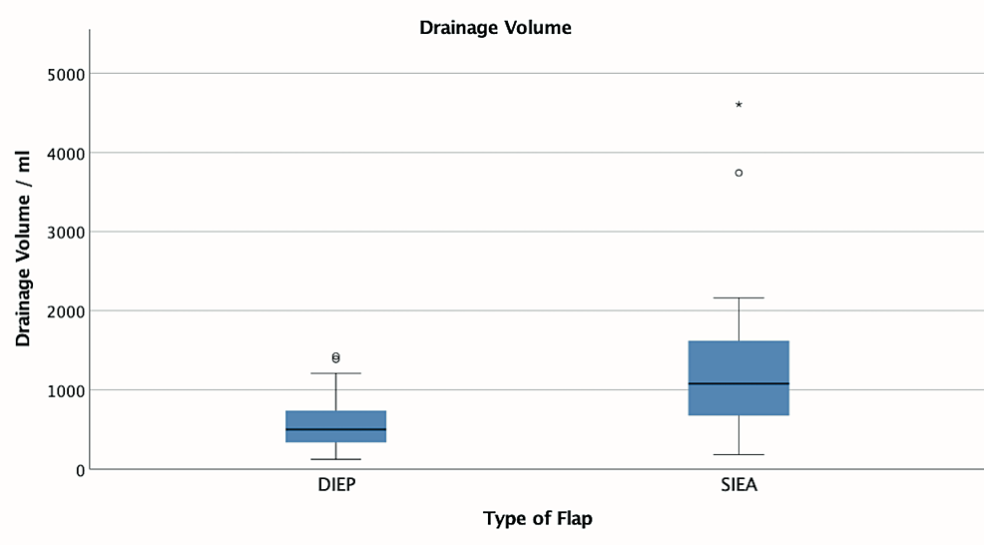
Total abdominal drain output Box plot showing cumulative output from abdominal drains including inpatient drainage and any drain outputs reported to the Plastic Surgery Dressings Unit by patients discharged with drains in situ. This box plot demonstrates the spread of data with median, upper and lower quartiles, and maximum and minimum excluding outliers. DIEP: deep inferior epigastric artery perforator, SIEA: superficial inferior epigastric artery

There was significantly higher median drain output following an SIEA harvest compared to only DIEP harvest (SIEA=1,078 mL, DIEP=500 mL, p<0.001), and this remained significant after controlling for BMI, flap weight, and age at operation (p=0.002) using regression analysis with multiple covariates (Figure 5). There was also a significant difference in the median day of drain removal (SIEA=11 days, DIEP=6 days, p=0.010), which remained significant after controlling for confounding variables (p=0.022).

Fourteen out of 25 patients who underwent an SIEA harvest were discharged with a drain, compared with two out of 25 patients who underwent only DIEP dissection. Patients who underwent an SIEA harvest were significantly more likely to be discharged with a drain in situ (odds ratio (OR)=14.6, 95% confidence interval (CI)=2.8203-75.9565, p=0.0014).

**Figure 6:**
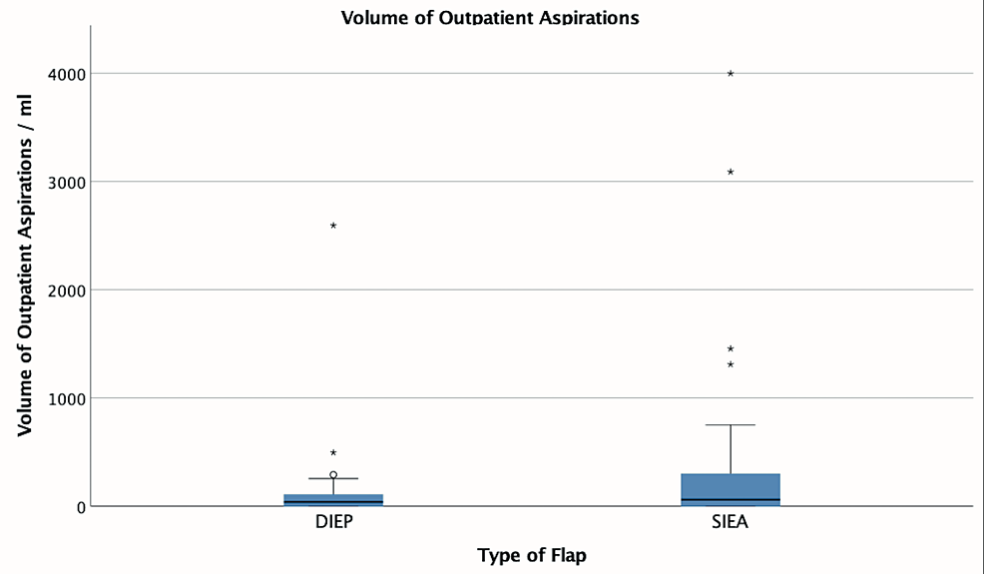
Box plot showing cumulative volume of outpatient aspirations following drain removal This box plot demonstrates the spread of data with median, upper and lower quartiles, and maximum and minimum excluding outliers. DIEP: deep inferior epigastric artery perforator, SIEA: superficial inferior epigastric artery

**Figure 7:**
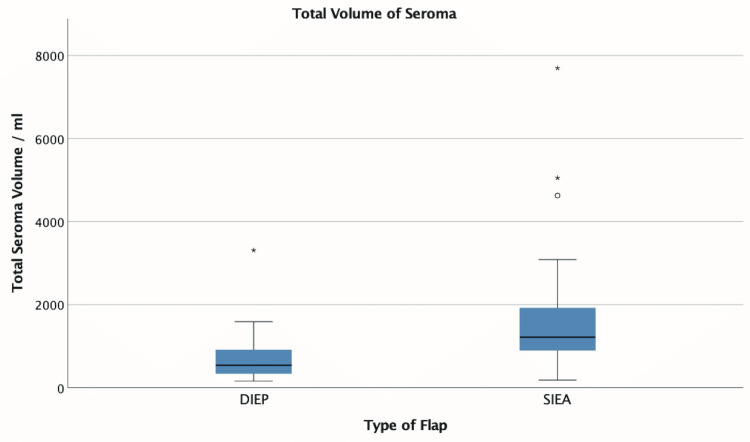
Box plot showing total abdominal donor site fluid, which is the sum of total drain output and subsequent seroma aspirations This box plot demonstrates the spread of data with median, upper and lower quartiles, and maximum and minimum excluding outliers. DIEP: deep inferior epigastric artery perforator, SIEA: superficial inferior epigastric artery

**Figure 8:**
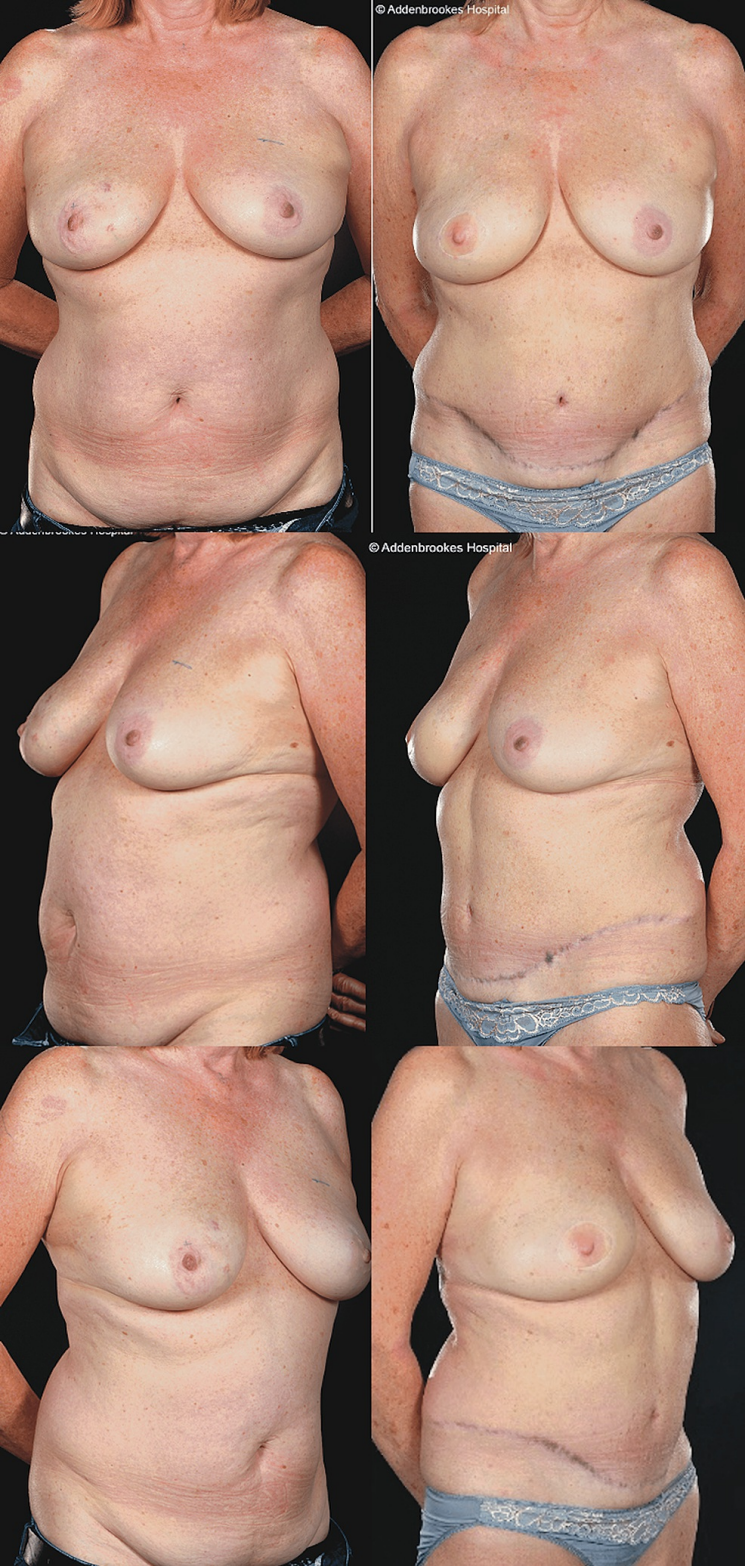
Unilateral SIEA flap This 58-year-old patient underwent a right skin-sparing mastectomy (675 g), sentinel lymph node biopsy, and immediate reconstruction with a left SIEA flap (891 g). Her preoperative and two-year postoperative clinical photographs show remarkable symmetry and well-maintained breast shape with excellent results from the nipple reconstruction and areolar tattooing. The inpatient drain output was 980 mL and she required one seroma aspiration of 115 mL postoperatively. SIEA: superficial inferior epigastric artery

**Figure 9:**
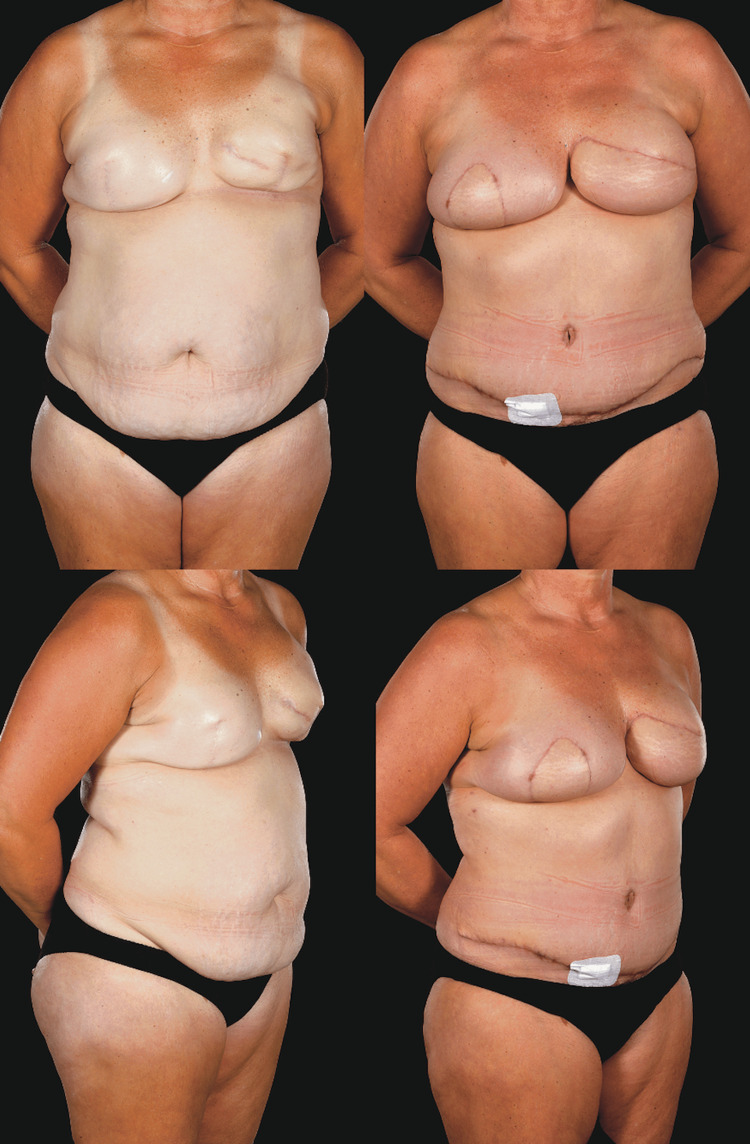
Bilateral SIEA flaps This 53-year-old patient underwent salvage (tertiary) breast reconstruction surgery to treat severe CC, poor cosmesis, and radiotherapy “burn” to her skin following previous implant-ADM delayed reconstruction elsewhere. She presented with pain, symptomatic CC, and poor aesthetic results, especially on the left previously irradiated side. The scar pattern was also challenging. The surgery comprised total capsulectomies, removal of Strattice ADM remnants, implant removal, and total autologous conversion to bilateral SIEA flaps. A larger skin paddle was needed on the left to replace the tight and scarred RT skin. She had a large seroma requiring multiple aspirations even at four months postoperatively as shown here by the dressing on the aspiration site. Preoperative BMI=30 kg/m^2^ SIEA: superficial inferior epigastric artery, CC: capsular contracture, ADM: acellular dermal matrix, BMI: body mass index, RT: radiotherapy

**Figure 10:**
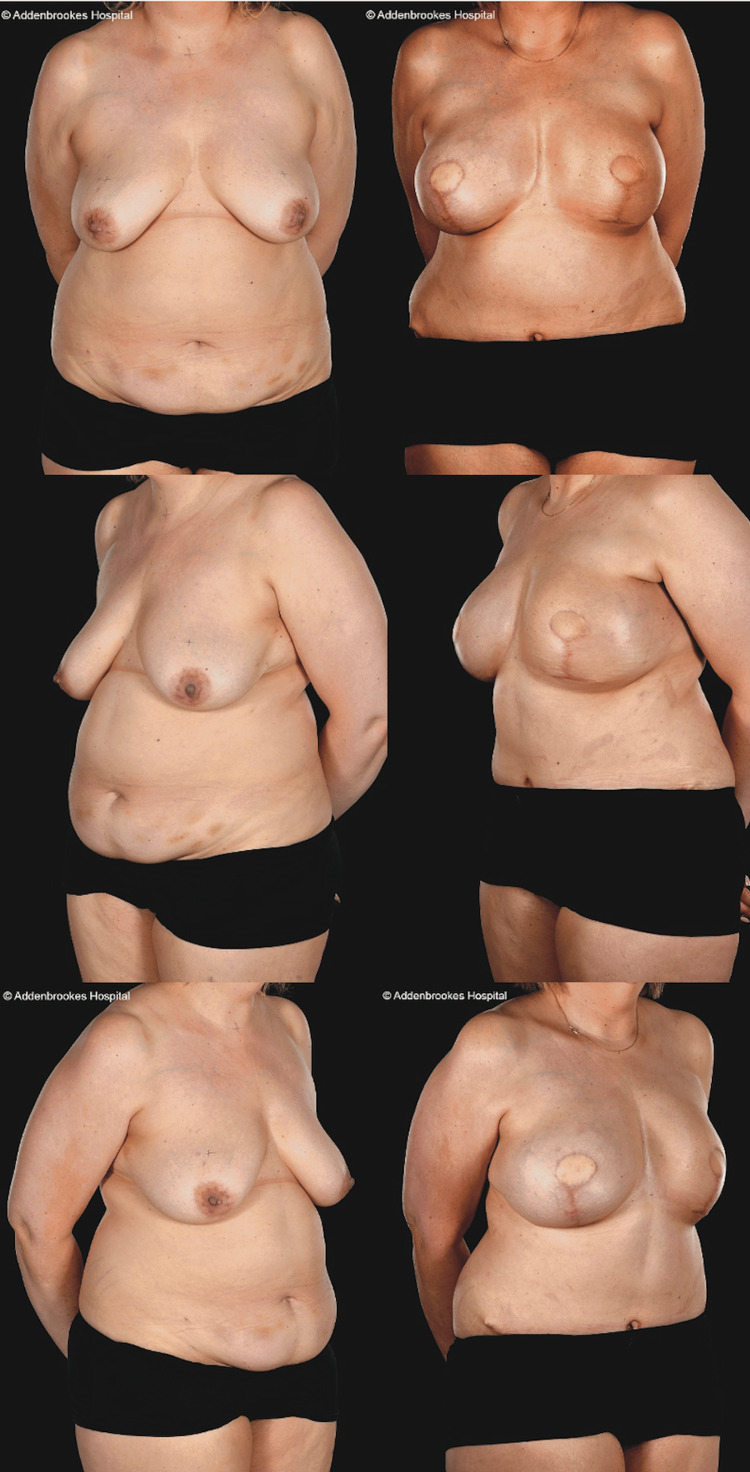
Bilateral SIEA and DIEP flaps This 44-year-old patient (BMI= 33.97 kg/m^2^) had bilateral breast cancers in mildly asymmetrical moderately sized (36DD/E bra cups) grade 2 ptotic breasts with poor skin quality and lost some weight preoperatively. She underwent bilateral LeJour pattern skin-reducing mastectomies, left axillary clearance, right sentinel lymph node biopsy with immediate bilateral abdominal free flap breast reconstruction (left SIEA weighing 1,028 g to reconstruct the right breast (mastectomy weight=722 g) and right DIEP flap (1,103 g weight) to reconstruct the left breast (mastectomy weight=840 g)). Pre- and six-month postoperative appearances (four months post-radiotherapy). She has not sought nipple reconstruction. She had large volumes of seroma fluid aspirated weekly or fortnightly for the first eight weeks: 480, 750, 610, 125, and 140 mL. The inpatient drain output was 1,630 mL. DIEP: deep inferior epigastric artery perforator, SIEA: superficial inferior epigastric artery, BMI: body mass index

Sixteen out of 29 (55%) patients had at least one outpatient seroma aspiration following SIEA flap harvest, compared with 19 out of 31 (61%) patients following only DIEP flap harvest. There was no significant difference in the median number (1 versus 1 aspiration, p=0.913) or volume (SIEA=60 mL, DIEP=37 mL, p=0.470) (Figure 6) of outpatient aspirations when compared using the independent samples median test. Similarly, length of hospital stay was not significantly different following SIEA harvest compared to DIEP (SIEA=7 days, DIEP=8 days, p=0.547). Total seroma volume, including both drainage and seroma aspirations, was significantly higher after SIEA harvest (SIEA=1,216 mL, DIEP=540 mL, p<0.001) (Figure 7). However, after controlling for confounding variables, flap type did not significantly predict the total seroma volume (p=0.054). Illustrative clinical cases of unilateral SIEA and bilateral SIEA flaps are shown in Figures 8-10.

## Discussion

SIEA flaps are preferred over DIEP flaps by some authors for breast reconstruction as, in suitable patients, they entail less invasive and quicker dissection with faster recovery and may reduce donor site morbidity while giving a reliable and aesthetically pleasing outcome [4,5,17,19] (Figures 8-10). However, SIEA flaps have unpredictable vascular anatomy, and the clinical impression of surgeons who perform SIEA flaps is that they lead to increased seroma formation with rates of up to 30%-50% (versus 5%-7% in DIEPs) in large centers [6,20,21].

Moradi et al. [18] showed a significantly increased total abdominal drainage of seven SIEA flaps compared with 28 DIEP flaps. Although this was a very asymmetric comparison (7 SIEAs versus 28 DIEPs), we have provided further quantitative evidence to support this with a larger number of SIEA harvests, showing that SIEA flap harvest is associated with increased drain output and longer time with a drain in situ. This translates to patients being more likely to be discharged with a drain in situ.

These results provide further considerations for surgeons when deciding which flap to use for breast reconstruction and how to counsel their patients preoperatively. This finding is significant because drains remaining in situ can put patients at risk of drain site infections and is an added postoperative inconvenience for patients, potentially reducing mobility. If inpatient abdominal drainage and time until drain removal are particular concerns for a given patient, then we suggest that DIEP flaps may be preferred over SIEA flaps in such scenarios. Interestingly, the number of outpatient visits is not significantly different between the two flap types, suggesting that difficulty getting to the hospital for outpatient seroma aspirations may not be a relevant consideration for patients when deciding between the two flaps. It is also important to note that often the decision to undertake an SIEA flap over a DIEP flap is made intraoperatively rather than preoperatively leading to potential difficulties with patient counseling.

We did not find a significant difference in inpatient stay between SIEA and DIEP flaps, largely due to the recent push within the NHS to discharge patients with abdominal drains in situ. In institutions where out-of-hours support for patients with abdominal drains is reduced or discharge with drains in situ is not possible, these patients may benefit from preferentially or deliberately carrying out DIEP flaps as opposed to SIEA to reduce the length of inpatient hospital stay.

We found no significant difference between the number or total volume of seroma aspirations carried out following drain removal in patients undergoing SIEA dissection. A possible explanation for this is that both SIEA and DIEP patients have their drains removed when drain output fell below the same set level. The likelihood of seroma formation at this point appears to be fairly comparable between SIEA and DIEP, as the drain outputs at this point in time are similar. The key difference shown in our study is that patients undergoing unilateral SIEA dissection take longer to reach this point than their unilateral DIEP counterparts. It is possible that when drains are removed based on other factors, the number or volume of seroma may differ between groups.

Limitations of the study are the lack of randomization of groups inherent in a long-term retrospective study, the use of sequential controls, and the inclusion of SIEA-DIEP bipedicled flaps or bilateral breast reconstructions with a DIEP flap on one side and an SIEA on the other. It is important to note that the SIEA flaps are not that commonly performed because of the vagaries of their anatomy, especially the artery. Therefore, an individual surgeon’s experience is likely to be limited. It should also be noted that not all of the SIEA flaps carried out by the senior author during this time period were included. This was due to insufficient data being present for some operations (19 patients), a well-known limitation of retrospective studies and paper records. Despite this, we are able to draw valid conclusions from our study where the results were statistically significant.

BMI and flap weight were shown to be significantly different between groups, and this may have contributed to increased donor site seroma. This was evident with the total seroma volume, where there was a significant difference in the total volume produced, but this did not remain significant after controlling for age, BMI, and flap weight. Flap weight was itself a significant predictor of the total volume of seroma (p=0.029). Despite this, we demonstrated that inpatient drainage and day of drain removal remained significant when controlling for flap weight.

The decision to perform an SIEA or DIEP flap takes into account many factors, particularly the intraoperative availability and reliability of the vascular pedicle. Increased abdominal fluid output following SIEA dissection is another factor for surgeons to consider when deciding what flap to raise, given the increased risks of infection and reduced mobility with prolonged time with drains and, in some centers, a potential for a longer hospital stay.

## Conclusions

Our retrospective case-control single-surgeon series clearly demonstrates the extent of increased drain output following SIEA harvest compared to DIEP harvest in breast reconstruction. Contrary to expectations, there does not appear to be an increase in volume or incidence of outpatient seroma aspirations with SIEA versus DIEP dissection, nor the total volume of seroma produced. However, an SIEA flap harvest was a significant predictor of increased drain output. This translated to a longer time before drain removal and patients being more likely to be discharged with their drains in situ.

## References

[1] Allen RJ, Heitland AS (2002). Superficial inferior epigastric artery flap for breast reconstruction. Semin Plast Surg.

[2] Taylor GI, Daniel RK (1975). The anatomy of several free flap donor sites. Plast Reconstr Surg.

[3] Holoyda KA, Simpson AM, Ye X, Agarwal JP, Kwok AC (2019). Immediate bilateral breast reconstruction using abdominally based flaps: an analysis of the nationwide inpatient sample database. J Reconstr Microsurg.

[4] Wu LC, Bajaj A, Chang DW, Chevray PM (2008). Comparison of donor-site morbidity of SIEA, DIEP, and muscle-sparing TRAM flaps for breast reconstruction. Plast Reconstr Surg.

[5] Chevray PM (2004). Update on breast reconstruction using free TRAM, DIEP, and SIEA flaps. Semin Plast Surg.

[6] Erdmann-Sager J, Wilkins EG, Pusic AL (2018). Complications and patient-reported outcomes after abdominally based breast reconstruction: results of the mastectomy reconstruction outcomes consortium study. Plast Reconstr Surg.

[7] Arnez ZM, Khan U, Pogorelec D, Planinsek F (1999). Rational selection of flaps from the abdomen in breast reconstruction to reduce donor site morbidity. Br J Plast Surg.

[8] Arnez ZM, Khan U, Pogorelec D, Planinsek F (1999). Breast reconstruction using the free superficial inferior epigastric artery (SIEA) flap. Br J Plast Surg.

[9] Chevray PM (2004). Breast reconstruction with superficial inferior epigastric artery flaps: a prospective comparison with TRAM and DIEP flaps. Plast Reconstr Surg.

[10] Holm C, Mayr M, Höfter E, Raab N, Ninkovic M (2008). Interindividual variability of the SIEA Angiosome: effects on operative strategies in breast reconstruction. Plast Reconstr Surg.

[11] Holm C, Mayr M, Höfter E, Ninkovic M (2007). The versatility of the SIEA flap: a clinical assessment of the vascular territory of the superficial epigastric inferior artery. J Plast Reconstr Aesthet Surg.

[12] Reardon CM, O'Ceallaigh S, O'Sullivan ST (2004). An anatomical study of the superficial inferior epigastric vessels in humans. Br J Plast Surg.

[13] Sadeghi A, Malata C (2013). Persistent seromas in abdominal free flap donor sites after postmastectomy breast reconstruction surgery: case reports and literature review. Eplasty.

[14] Granzow JW, Levine JL, Chiu ES, Allen RJ (2006). Breast reconstruction with the deep inferior epigastric perforator flap: history and an update on current technique. J Plast Reconstr Aesthet Surg.

[15] Tomouk T, Mohan AT, Azizi A, Conci E, Brickley EB, Malata CM (2017). Donor site morbidity in DIEP free flap breast reconstructions: a comparison of unilateral, bilateral, and bipedicled surgical procedure types. J Plast Reconstr Aesthet Surg.

[16] Menn Z, Spiegel A (2012). The superficial inferior epigastric artery (SIEA) flap and its applications in breast reconstruction. Breast reconstruction.

[17] Spiegel AJ, Khan FN (2007). An Intraoperative algorithm for use of the SIEA flap for breast reconstruction. Plast Reconstr Surg.

[18] Moradi P, Durrant C, Glass GE, Askouni E, Wood S, Rose V (2011). SIEA flap leads to an increase in abdominal seroma rates compared to DIEP flap for breast reconstruction. Eur J Plast Surg.

[19] Munhoz AM, Pellarin L, Montag E (2011). Superficial inferior epigastric artery (SIEA) free flap using perforator vessels as a recipient site: clinical implications in autologous breast reconstruction. Am J Surg.

[20] Hamdi M, Blondeel PN (2006). The SIEA flap in breast reconstruction. Surgery of the breast: principles and art.

[21] Spiegel AJ, Eldor L (2018). Flap breast reconstruction. Plastic surgery: breast.

